# Chemopreventive activity of celastrol in drug–resistant human colon carcinoma cell cultures

**DOI:** 10.18632/oncotarget.25014

**Published:** 2018-04-20

**Authors:** Helena Moreira, Anna Szyjka, Kazimierz Gąsiorowski

**Affiliations:** ^1^ Department of Basic Medical Sciences, Wroclaw Medical University, Borowska 211, 50-556 Wrocław, Poland

**Keywords:** celastrol, colon cancer, multidrug resistance, cancer stem cells

## Abstract

Celastrol (tripterine) a pentacyclic triterpenoid extracted from the roots of *Tripterygium wilfordii* Hook f., exhibits potent antioxidant and anti-inflammatory activity and also exerts important anti-cancer effects, as induction of apoptosis and lowering the level of drug resistance of several cancers.

Increased level of cellular resistance to cytostatic drugs is typical for colorectal cancers, and largely determines the failure of chemotherapy for this tumor.

The purpose of our research was to evaluate the chemopreventive effect of celastrol on cultures of colon cancer cells resistant to doxorubicin (LOVO/DX). With the use of flow cytometry we have shown that celastrol reduces the cell size of the SP (side population; subpopulation of cancer cells enriched with cancer stem cells), increases frequency of apoptosis and binds to Pgp protein in cell membranes inhibiting its transport function. The inhibition of the Pgp transport function has been shown to increase the accumulation of rhodamine-123 and standard cytostatic- doxorubicin in LOVO/DX cells.

Our results prove that celastrol exhibits significant chemopreventive and chemosensitizing activities on drug resistant colon cancer cells. Celastrol appears to be a good candidate for adjuvant medicine that can improve the effectiveness of standard cytostatic therapy in humans.

## INTRODUCTION

Colon cancer is the third most common form of cancer and the second leading cause of cancer-related death in western countries [1]. Despite of existing advanced therapies, i.e. targeted therapy, the 5-year survival rate is still barely 12.5% [2]. The primary reason for treatment failure is a marked resistance of colon tumor cells to chemotherapy. In patients with metastatic cancer, that is considered incurable, the occurrence of drug resistance is more than 90% [2, 3]. Therefore, the novel treatment strategies are needed to overcome or evade the drug-resistance of colon cancer.

In order to improve the outcomes of colon cancer treatment, attention is paid to the role of cancer stem cells (CSCs) in the development of drug resistance status of the tumor. Cancer stem cells (CSCs) are considered to be responsible for tumor initiation, drug and radiation resistance, invasive growth, metastasis, and tumor relapse, which are the main causes of cancer-related deaths [4]. Many authors assume that CSCs are master regulators in the process of drug resistance acquisition [5–8]. CSCs play an important role in recurrence of colon cancer following chemotherapy, because of their resistance to cytostatic drugs and the ability to unlimited proliferation and self-renewal [9]. The main mechanism of the development of colon cancer cells drug-resistance is the overexpression of glycoprotein P (P-gp). This protein, encoded by the *ABCB1* [multidrug resistance protein 1 (MDR1)] gene, belongs to the family of ATP-dependent transporters (ABC transporters), which actively removes the chemotherapeutic drugs from cancer cells [10, 11].

It was documented in the literature, that various natural compounds of plant origin are potent P-gp blocking agents, reducing cancer cell drug resistance [12, 13]. They also inhibit the function of tumor stem cells [14, 15] and exert a number of other beneficial chemopreventive effects [16, 17].

Among natural, plant-derived compounds celastrol, also known as tripterine, obtained from roots of *Tripterygium wilfordii* Hook.f. and *Celastrus regelii*, L. exhibits potent antioxidant and strong anti-inflammatory activities; in the near future these effects of celastrol will be used clinically for treatment of chronic inflammatory disorders, such as rheumatoid arthritis, and neurodegeneration of Alzheimer's type and amyotrophic lateral sclerosis [18–22]. Celastrol also exerted significant anti-neoplastic effect in various cancer models, e.g. cervical, prostate, gastric, breast and colon [23, 24]. It markedly reduced the level of drug resistance of various human cancer cell cultures [25–29]. For instance, in cultures of human doxorubicin resistant myelogenous leukemia - K562/A02 celastrol increased leukemic cell sensitivity to cytotoxic agents, decreased the P-gp expression and significantly elevated intracellular content of doxorubicin in leukemic cells [30].

In this paper, we have examined chemopreventive effect of celastrol on drug resistant colon cancer cells, including its impact on functional/transporter activity of the P-gp, on frequency of apoptosis and necrosis in colon cancer cell cultures, as well as on size of the SP cells subpopulation (subpopulation of cancer cells assumed to be enriched with cancer stem cells; CSCs).

## RESULTS

**Figure 1:**
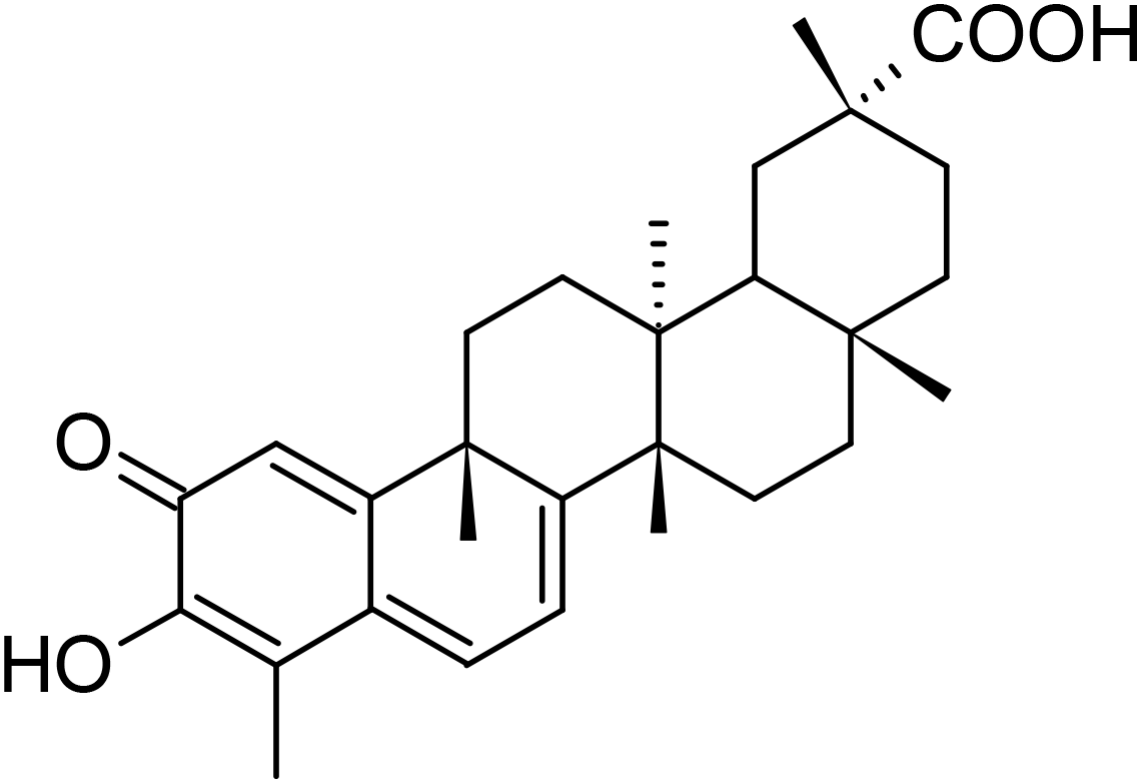
Chemical structure of celastrol

Chemical structure of celastrol is given in the scheme in Figure 1.

Celastrol is cell-permeable pentacyclic triterpenoid (MW 450.6), soluble in 100% ethanol, DMSO (insoluble in water), which belongs to the family of quinone methides.

### Impact of celastrol on apoptosis and necrosis in LOVO/DX cell cultures

**Figure 2:**
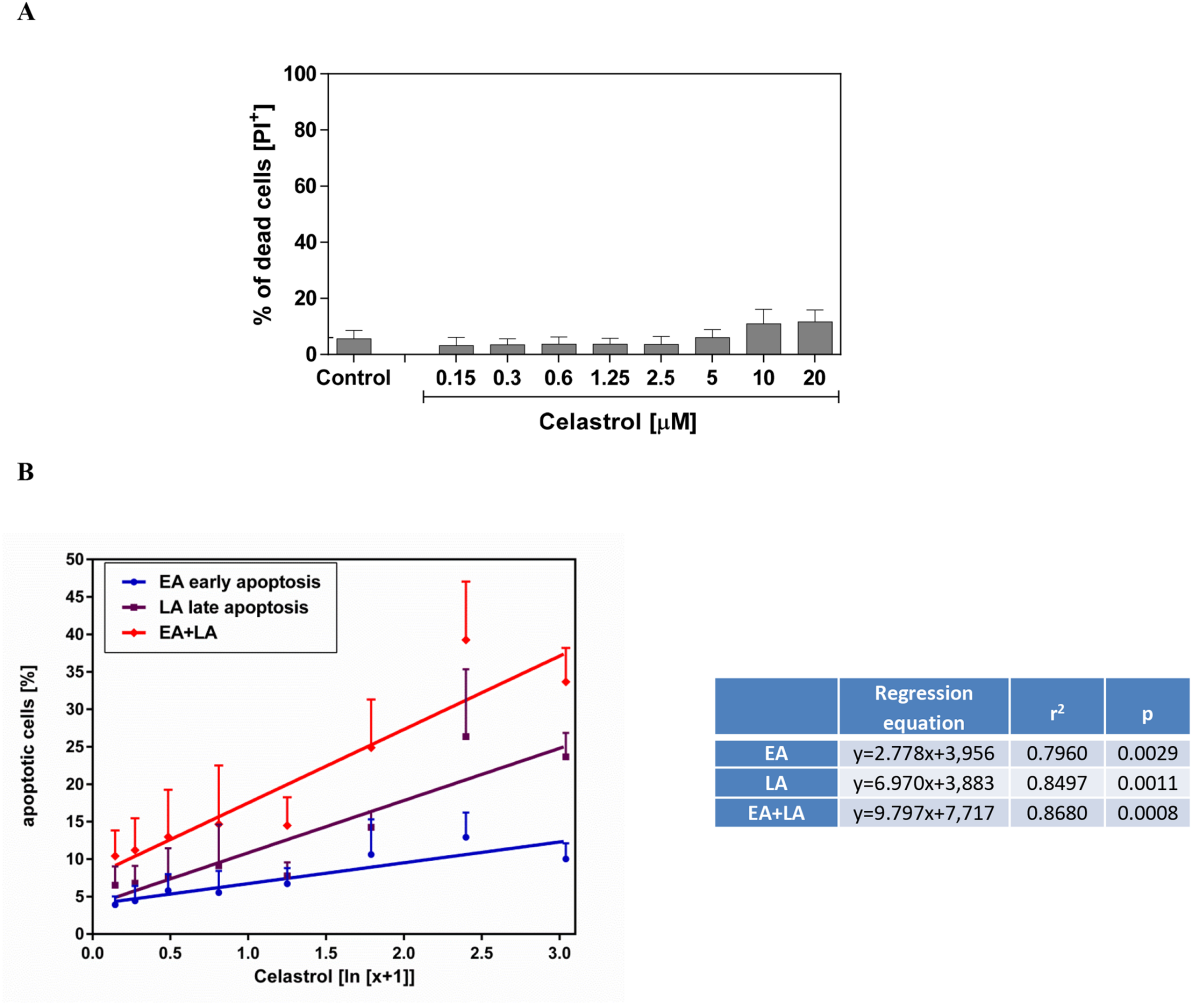
Impact of celastrol on frequency of necrotic (A) and apoptotic (B) cell in LOVO/DX cell cultures The cells (5×10^5^/ml) were incubated [4h, 37°C] in the absence (control) or presence of celastrol, and then double stained with Annexin V-FITC and PI fluorescent probes (FITC Annexin V Apoptosis Detection Kit I BD Biosciences). The result are presented as a percentage of dead cells (PI^+^), early apoptotic (EA) cells (Annexin V-FITC^+^ and PI^−^) and late apoptotic (LA) cells (Annexin V-FITC^+^ and PI^+^). The dose-dependent effects were calculated with regression equations (mean±SD; n=4).

First, we evaluated the effects of celastrol on the viability of the LOVO/DX cells using a membrane impermeant dye - propidium iodide (PI). As shown in Figure 2A, the treatment of LOVO/DX cells with celastrol for 4h did not affect cell viability. The percentage of death cells (PI^+^ cells) remained near control level (3-6%) at almost all tested concentrations of celastrol (ranging from 0.15 to 5.0μM). Only very small 5-6% increase in the proportion of death cells was observed at 10 and 20μM of celastrol, however it was statistically non-significant.

In parallel, we studied whether celastrol induces apoptosis in LOVO/DX cells. For this purpose, we used Annexin V-FITC, fluorescent probe detecting phosphatidylserine expression on apoptotic cells, and PI. The Annexin V-FITC/PI double staining allowed us to discriminate between early and late apoptotic cells. Celastrol was found to induce apoptosis in LOVO/DX cells in dose-dependent manner (Figure 2B). Proapoptotic effect of celastrol was observed after 4 hours incubation with cells. As may be seen in Figure 2, celastrol triggered an increase in the number of both early (Annexin V-FITC positive and PI negative) and late (Annexin V-FITC positive and PI positive) apoptotic cells. The regression equations given in Figure 2B indicate that the observed effect on late apoptosis was 2.5 times strongest than that on early apoptosis. Taken together, celastrol induce up to 38% increase in proportion of total apoptotic cells (early+late).

### Influence of on DOX and Rod123 content in LOVO/DX cells

**Figure 3:**
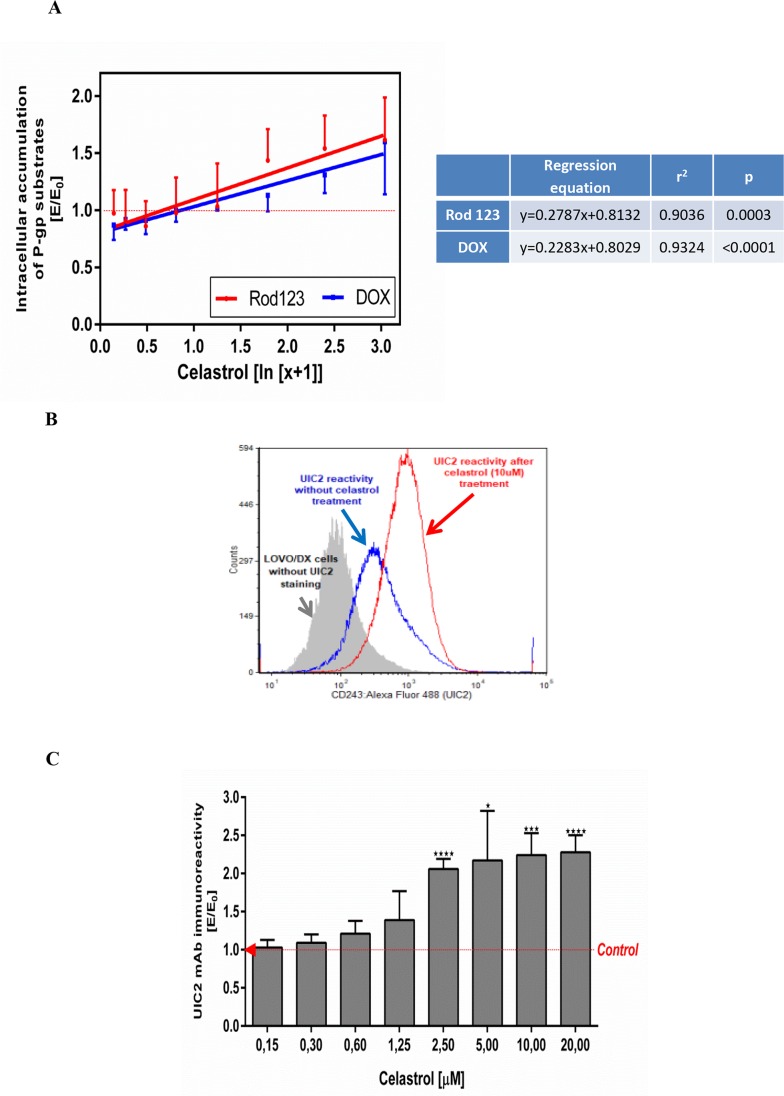
Influence of celastrol on P-gp structure and transport function in LOVO/DX cell cultures **(A)** Accumulation of doxorubicin (DOX) and rhodamine 123 (Rod-123) in colon cancer cells. The cells (5×10^5^/ml) were pre-incubated (5min) with celastrol following by incubation with DOX (3μM) or Rod-123 (5μM) [1h, 37°C]. The cells-associated fluorescence was evaluated by flow cytometry. The result obtained in the presence of celastrol (E) were compared to the relevant control culture (E_0_), i.e. LOVO/DX culture (without celastrol) and expressed as E/E_0_ ratios. The dose-dependent effects were calculated with regression equations. (mean±SD; n=4 (DOX) and n=8 (Rod-123)). **(B)** Representative histograms of the UIC2 mAb immunoreactivity with Pgp in LOVO/DX cell cultures treated with 10μM of celastrol (red line) or with diluent only (DMSO, blue line). The cells were incubated with celastrol [10min., 37°C] and then stained with anti-CD243 Alexa Fluor 488 (UIC2 mAb) celastrol [15min., 37°C]. The cells-associated fluorescence was evaluated by flow cytometric analysis **(C)** Immunoreactivity of the UIC2 mAb with Pgp after LOVO/DX cell culture treatment with the range of celastrol concentrations. Results are expressed as E/E_0_ ratios (mean±SD, n=4; ^*^p<0.05, ^***^p≤0.0001), where MFI (mean fluorescence intensity) of the UIC2 mAb estimated in cell cultures incubated with celastrol (E) were compared to MFI of the UIC2 mAb of cells cultured in the presence of DMSO (E_0_).

To assess whether celastrol can reduce the drug resistance of LOVO/DX cells we examined its effects on the intracellular accumulation of doxorubicin (DOX), an anthracycline antitumor antibiotic. As was shown in Figure 3A, celastrol increased DOX accumulation in LOVO/DX cells in the dose-depended manner and the effect reached the level of statistical significance in the concentration of celastrol in cell culture in the range of 5-20μM (1.13-1.60 fold increase of DOX content in cells). Since DOX is known to be P-gp protein substrate, these results suggest that celastrol could inhibit the transport activity of this MDR protein. In order to confirm this effect of celastrol we have performed a study on the accumulation of rhodamine 123 in LOVO/DX cells. Rhodamine 123 (Rod 123) is a fluorescent probe commonly used to measure the functional activity of P-gp. The results (Figure 3A) proved that celastrol increased Rod 123 accumulation in LOVO/DX cells and the level of Rod 123 content in the cells was markedly higher than that of DOX (up to 30% higher in celastrol 5μM). These results show that celastrol inhibits the removal of xenobiotics (including cytostatics) from LOVO/DX drug resistant cell lines.

### Celastrol binding to Pgp protein

Because the main mechanism responsible for the removal of cytostatic drugs from the cells is the transport function of the membrane protein Pgp, we have evaluated the binding of celastrol to the protein Pgp in the “UIC2-shift assay”. This assay is based on the increased immunoreactivity of a functional anti-P-gp monoclonal antibody (UIC2 mAbs) in the presence of P-gp modulators/inhibitors under physiologic conditions. Flow cytometric analysis shown in Figure 3B reveals that celastrol (10μM) caused increased binding of UIC2 mAbs to LOVO DX cells. The immunoreactivity of UIC2 mAbs rised along with increased celastrol concentrations in the cell culture medium (Figure 3C). Significant, more than 2–fold increase was observed at 2.5, 5, 10 and 20μM of the celastrol final concentrations.

### Effect of celastrol on size of the SP cell subpopulation

Impact of celastrol on size of the SP cells subpopulation in LOVO/DX cell cultures was evaluated with the Hoechst 33342 efflux assay. Side Population (SP) has been identified in several cancers as subpopulation significantly enriched with cells exhibiting some stem-like characteristics, for instance, an overexpression of MDR transporters, as Pgp protein. This subpopulation of cancer cells could be detected by flow cytometry, based on their capacity to efflux the DNA-binding dye Hoechst 33342.

**Figure 4:**
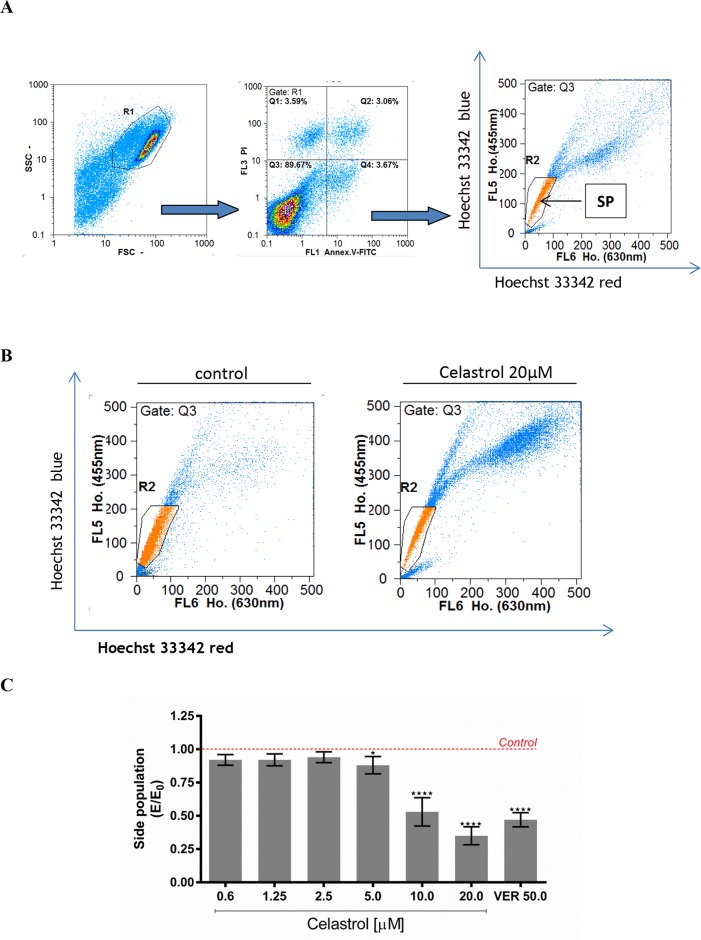
The effect of celastrol on cell size of the SP subpopulation in LOVO/DX cell cultures **(A)** A gating strategy used to analyze only singlet viable cells. The cells were pre-incubated with celastrol for 5-10 minutes with subsequent incubation with DNA-binding dye Hoechst 33342 (Ho.) [5μg/ml] for 90 minutes at 37°C. Then the cells were stained with Annexin V-FITC and PI for exclusion of dead and apoptotic cells from the analysis. The cells-associated fluorescence was evaluated by the means of flow cytometry. SP (Side Population) is defined as subpopulation of cells that show the lowest Ho. content (a low-Ho.fluorescence “tail” in dual wavelength of fluorescence emission: Hoechst red [630nm]and Hoechst blue [455nm]). **(B)** Representative cytograms of cell size of the SP subpopulation in the presence of celastrol [20μM] or vehicle-DMSO. **(C)** Cell size of the SP subpopulation after treatment of LOVO/DX cultures with the range of celastrol concentrations. Results are expressed as E/E_0_ ratios, where E = % of SP cells in cultures incubated with celastrol and E_0_ = % of SP cells in cultures incubated with diluent -DMSO (mean ±SD, n=6 ; ^*^p<0.05, ^***^p<0.0001).

Figure 4A shows cytometric analysis of size of the SP cells subpopulation in control LOVO/DX cultures (without celastrol). As can be seen in the Figure 4B, the SP fraction in the celastrol (20μM) treated cells was markedly lower than that of the control cultures. Celastrol caused decrease of SP subpopulation size at all concentrations tested, however a marked decrease was observed at 10 and 20μM of celastrol (Figure 4C). This effect was of similar strength as that of verapamil (50μM), the well-known P-gp inhibitor.

### Expression of ALDH1 in LOVO/DX cells after incubation with celastrol

The frequency of ALDH1^+^ cells was assessed in the entire population of LOVO/DX cells and in the SP subpopulation. For this purpose, we used Hoechst 33342 staining to identify SP cells and ALDEFLUOR™ assay to detect ALDH1 activity. We found very small number (about 2,40%) of cells highly expressing ALDH1 in cell culture, and only a little overlap between those cells and the SP population (±1% of ALDH+ cells were SP cells) was noticed. Incubation of the cell cultures for 30min with celastrol resulted in a decrease in the expression of ALDH1 (mean fluorescence intensity, MFI) in the entire population of the test cells as shown in Table 1.

**Table 1:** Effect of celastrol on ALDH1 fluorescence in LOVO/DX cells with ALDEFLUOR™

	ALDEFLUOR™ (Median Fluorescence Intensity)
**Control cells**	20,58
**Celastrol [1μM]**	18,31
**Celastrol [10μM]**	16,14

LOVO/DX cells were pre-treated with celastrol for 30 minutes and then incubated with Hoechst33342 for 90 minutes. Following incubation, cells were stained with ALDEFLUOR™ and analyzed by flow cytometry

The reduction in fluorescence intensity (ALDH1 expression level) in the LOVO/DX culture was proportional to the celastrol concentration: by 11% (1 μM) and 22% (10 μM) compared to the relative control sample of cells not incubated with celastrol.

### Statistical evaluation of the results

Comparison of the celastrol impact on LOVO/DX cell cultures in six tests was done using a Kruskal-Wallis test with Dunn's post hoc test and a Spearman's rank correlation test. The results of the calculation are given in Table 2.

**Table 2:** Statistical analysis of the results obtained with 6 tests evaluating the effect of the celastrol on LOVO/DX cell cultures

size of the SP cell subpopulation *versus:*	A		B	
	Kruskal-Wallis test:	Dunn's post hoc test	Spearman's rank correlation test	
		p	r_S_	p
**apoptosis frequency**	H = 20.6; N = 6; p = 0.0010	0.0067	- 0.928	0.022
**necrosis frequency**		0.9999; *NS*	- 0.868	0.042
**celastrol binding to Pgp**		0.0055	- 0.899	0.028
**intracellular content of Rod 123**		0.3951; *NS*	- 0.899	0.028
**intracellular content of DOX**		0.6599; *NS*	- 0.899	0.028

A – Kruskal-Wallis test with Dunn's post hoc test, B – Spearman's rank correlation test.

As can be seen in column A of Table 2 strong statistically significant differences were calculated between the effect of celastrol in 6 tests (p = 0.0010). However, when compare the results of individual tests to the effect of celastrol on size of SP cells subpopulation (with Dunn's post hoc test), only statistically significant differences of celastrol impact on apoptosis and on its binding to Pgp could be estimated (p = 0.0067 and p = 0.0055, respectively).

The correlations between the results obtained with 6 tests were calculated by comparing the results of each test to the size of SP cells subpopulation, and correlation coefficients and p values were given in column B of Table 2. All the correlation coefficients in Table 2 B were highly negative and were statistically significant. The highest negative correlation coefficient was calculated for apoptosis incidence in LOVO/DX cultures incubated with celastrol; in these cultures the decrease of cell size of the SP subpopulation was accompanied by a marked increase in the frequency of LOVO/DX cell apoptosis. Additionally, calculated correlation coefficients between celastrol binding to Pgp and a content of Rod 123 and of DOX in LOVO/DX cells show high correlation between compared data: r_S_ = 0.976, p = 0.004 and r_S_ = 0.905, p = 0.005 (data not included in the paper).

## DISCUSSION

Celastrol, a plant-derived pentacyclic triterpenoid, exhibits anticancer effects by affecting the different signaling pathways of tumor cells [13, 23]. It is highly reactive to nucleophilic thiol groups of cysteine residues and forms covalent adducts with numerous proteins, affecting their function. It is assumed that the main mechanism of its anticancer action is due to the change of function of the important protein in tumor cells [23, 31]. Celastrol has been shown to exert an anti-cancer effect against many cancers *in vitro* including pancreatic, gliomas, prostate, breast, gastric and colon cancer and also several leukemia cell lines [23, 24]. However, the effect of celastrol on the condition and mechanisms of chemo-sensitivity of colorectal cancer cells has not been studied in detail. In our current work we show the effect of celastrol on chemoresistance status of the LOVO/DX - multidrug resistant human colon cancer cell line.

The chemopreventive activity of anti-tumor preparations includes their effect on increased cancer cell death. Up to now, different type of cell death have been distinguished, including apoptosis, autophagy, necrosis and paraptosis [32]. It is well established that celastrol is a potent pro-apoptotic agent and can promote apoptosis in various cancer cell cultures [33]. Recent reports indicate that celastrol may increase tumor cell death not only by apoptosis but also by other types of dying, e.g. by paraptosis and autophagy, as was demonstrated in the breast and colon cancer cell cultures [34], HeLa, A549, PC-3 [35] and osteosarcoma cells [36]. In our current work we have analyzed whether celastrol is equally potent in induction necrosis and apoptosis in LOVO/DX cell cultures. Our results revealed that short (4-hours) exposures of LOVO/DX cell cultures to celastrol result in significant increase in apoptotic cell frequency. The rate of late apoptosis was 2.5 times higher than that of early apoptosis indicating that celastrol produce quick apoptotic changes in those cells. However, we did not observe the effect of celastrol on frequency of necrotic form of cell death in LOVO/DX cell cultures These findings suggest that in cultures of drug-resistant colon cancer cell celastrol exerts its cytotoxic effect by induction of suicide cell death programs and not by unprogrammed, simple necrotic cell death.

The main reason for the failure of colon cancer treatment is the high level of resistance of this tumor to cytotoxic drugs. Colon tumor cells chemoresistance can be both intrinsic or acquired after chemotherapeutic cure. There are several mechanisms that contribute to the overall resistance of colorectal cancer, including overexpression of gluthathione S-transferase-π, topoisomerase II and P-glycoprotein (P-gp) [10, 37]. P-glycoprotein belongs to the large family of ABC (ATP-dependent) active transporters and works as a transmembrane efflux pump for xenobiotics and various cytotoxic drugs. In normal colon cells, P-gp is constitutively expressed and play the role in controlling of oral availability of many substance [38]. Colon carcinoma cells retain the capacity to express P-gp and can maintain it throughout all stages of colon tumor progression [10]. Increased expression and efflux function of P-gp in tumor cells results in reduction of intracellular drug concentrations with consequent decrease in the cytotoxicity of a wide range of cytotoxic drugs e.g. doxorubicin. Therefore, inhibition of P-gp function leads to chemosensitization of cancer cells via increasing accumulation of anticancer drug, and thus to overcoming MDR [39]. The large number of chemical and naturally occurring compounds are known as P-gp inhibitors, including cyclosporines, calmodulin inhibitors, indole alkaloids, coronary vasodilators, quinolines, hormones, calcium channel blockers, flavonoids, stilbenes, coumarins, saponins, alkaloids and also terpenoids [39]. Various terpenoids can modulate P-gp transport more or less efficiently, depending on the structure [40]. Recently, Munoz-Martinez F. *et al.* demonstrated that sesquiterpenes from Celastraceae plant were able to potently reverse cellular MDR by blocking specifically human P-gp without affecting the activity of MRP1, MRP2 and BCRP proteins [41, 42]. In this work we demonstrated that in P-gp expressing LOVO/DX cells, celastrol was able to significantly increase the intracellular level of P-gp substrates: rhodamine 123 and doxorubicin. To prove that the observed effect was due to direct interaction of celastrol with P-glycoprotein we used highly specific anty-P-gp (UIC2) monoclonal antibody. UIC2 mAb is specific for a conformation-sensitive P-gp external epitope (CD243) [43]. It was well established, that binding of substrate or inhibitor to P glycoprotein at physiological conditions causes changes in protein conformation making CD 243 better accessible for UIC2 antibody [44, 45]. We showed, that in the presence of celastrol the UIC2 antibody reactivity with LOVO/DX cells was strongly increased, even 2-fold compared to control cells. This finding clearly indicates that celastrol inhibits functionality of P-gp, by direct interacting with this protein.

Colon cancer resistance to chemotherapy is linked to the content of cancer stem cells (CSCs) which exist in an SP (Side Population) of cancer cell mass [4, 46]. Cells within SP subpopulation share many characteristics with normal stem cells, including overexpression of multidrug resistant proteins: P-gp, MDR1 and BCRP [47, 48], thus several P-gp inhibitors, that bind to P-gp protein, change its conformation, thereby inhibit drug efflux from CSCs [15]. The SP cells subpopulation could be identified *in vitro*, with flow cytometry analysis, based on their enhanced efflux of Hoechst 33342 dye through MDR transporters [49–51]. Various human cancer cell lines contain small but clearly countable cell size in the SP subpopulation [52, 53]. Our results show that size of the SP cells subpopulation was almost 7 fold greater within cultures of the LOVO/DX (doxorubicin resistant cell line) than in the LOVO/WT (cells ordinary sensitive to cytostatics), but incubation with celastrol caused significant reduction of size of the SP cells subpopulation within the LOVO/DX cell cultures. For instance, in the presence of celastrol at 20μM, the size of the SP cells subpopulation was reduced by 65% when compared to the control cultures (without celastrol) and the effect was markedly stronger than that of the standard Pgp inhibitor - verapamil (decrease by 55%).

Recent data indicate that CSCs population in tumors could vary over time and that tumor microenvironment exerts a dominant role in determining CSCs number and stemness features in tumor cell mass [7]. Interaction between cancer stem cells and adjacent stromal cells within the CSC niche, a response to signals received from neighboring tumor cells could induce both differentiation of stem cells (loss of stemness) and dedifferentiation of non-tumorigenic cells of tumor cell mass (gain of stemness features) [7, 8]. As CSCs number in tumor cell mass changes dynamically according to the signals from neighboring cells, the stemness of cancer cells should be perceived rather as the functional state of the cells and not as a specific cell phenotype [8, 54].

Statistical analysis of the results obtained in our work showed high negative correlation between reduced size of the SP subpopulation and associated increase in apoptosis, enhanced celastrol-glycoprotein P binding accompanied with higher accumulation of the standard cytostatics (doxorubicin) in cancer cells. Since the abundance of SP subpopulations, chemosensitivity of cancer cells to cytostatic drugs and susceptibility to apoptosis; all of them are important functional attributes of CSCs, so it can be assumed that celastrol has a beneficial effect on some of the features of CSCs and it is desirably reducing the level of stemness in the LOVO/DX culture.

As celastrol inhibits the expression of NF-κB [59–61], the effects we observed, including the increase in apoptosis in LOVO/DX cell cultures, inhibition of Pgp transport function - increase in intracellular fluorochrome accumulation (Hoechst 33342, Rod-123), could be explained with a decrease in the function NF-κB by the celastrol. In conclusion, our results indicate the significant chemopreventive effect of celastrol on the cultures of drug-resistant colon cancer cells. Literature data show that in human tumors, activation of NF-κB leads to an increase in the expression of the MDR1 gene and the product of this gene - efflux protein Pgp [62, 63], and inhibition of NF-κB activity results in a decrease in the transport function of Pgp [64]. NF-κB inhibits tumor cell apoptosis, both the course of the receptor pathway and the mitochondrial pathway, and the mechanisms of this action are very complex and result mainly from the function of this transcription factor in the regulation of gene expression in the pathways of apoptosis [65–67]. Because increased gene expression of multidrug resistance proteins and reduced apoptotic response are important reasons for the development of acquired multidrug resistance of tumor cells, attempts to inhibit the function of NF-kB are a promising direction for increasing the sensitivity of tumor cells to cytostatic drugs [65, 66, 68, 69]. Celastrol seems to be a good candidate for chemosensitizing adjuvant medical preparation in cytostatic chemotherapy of cancer, in particular it could enhance the response to the standard cytostatic drugs of these tumors, which contain a large subpopulation of drug-resistant cells.

The rapidly growing number of publications shows that the activity of the NF-κB transcription factor in regulating the expression of many genes also relates to the function and phenotype of tumor stem cells [67, 70, 71]. It has been shown that in the subpopulation of cancer stem cells NF-κB significantly increases the expression of genes of cellular proteins associated with chemoresistance and invassiveness of cancers, among which intensifies the expression and function of P-glycoprotein, reduces cell susceptibility to apoptosis, enhances repair of DNA damage in cancer cells and increases the ability of new migration and metastasis [65, 66, 68, 69]. NF-κB activation pathways (both canonical, non-canonical and alternative pathways) are very complex, multi-component [70, 71] and often have a different effect on the phenotype and function of cancer cells. For example, the gene expression and function of ALDH aldehyde dehydrogenase proteins in human ovarian cancer stem cells is shown to be reduced when the RelB subunit is inhibited, while the inhibition of the RelA component of the NF-κB has no effect on ALDH expression [70]. In human breast cancer cell lines the content of cancer stem cells was correlated with high expression of NF-κB –inducing kinase (NIK) and was also related to elevated expression of ALDH1A1 gene [71]. The authors point out that NF-κB sustains diverse cancer stem cell phenotypes via distinct classical and alternative signalling pathways, and this feature of the protein is important to understand cancer biology, including disease recurrence and cancer susceptibility to chemotherapy [70, 71]. Possible pharmacological intervention in the pathways of NF-kB activation may have different effects on various components of the activation of this protein and on the growth of tumor cells. For example, in our experiments there was a decrease in the level of expression of ALDH1 under the influence of celastrol, in combination with inhibition of proliferation, increase in LOVO/DX apoptosis in cultures. Therefore, it seems that the assessment of significant functional features of neoplastic growth is more clinically applicable than attempts to identify closely the phenotypic features of cancer stem cells that can be changed in a given tumor, depending on the cellular and extracellular context. However, at the present stage of knowledge, the NF-κB is a very promising target for cancer pharmacotherapy and targeted disruption of this pathway may profoundly impair the adverse phenotype of cancers, also of cancer stem cells and could significantly enhance chemosensitivity of tumors. Further studies on the influence of the NF-kb inhibitor – celastrol - on tumors, including cancer stem cells, should explain the role of this triterpene in inhibiting the growth of tumors and will help to define the supporting role of celastrol in different schemes of standard cytostatic treatment.

## MATERIALS AND METHODS

### Cell line and culture conditions

The doxorubicin-resistant colon adenocarcinoma cell line (LOVO/DX) wasderived from the original drug-sensitive LOVO cell line by 3-months cultivation in the presence of low concentration of doxorubicin. Cells were cultured in DMEM F12 medium (Dulbecco's Modified Eagle Medium: Nutrient Mixture F-12) ( Lonza, Basel, Switzerland) supplemented with 10% fetal bovine serum (FBS) (Lonza, Basel, Switzerland), 2mM L-glutamine (Lonza, Basel, Switzerland) and 25mg/ml of gentamicin (Lonza, Basel, Switzerland) at 37°C in a humidified atmosphere with 5% CO_2_. The cells were subcultured twice a week using TrypLE™ Express (GIBCO, Waltham, MA, USA).

### Drugs and reagents’ solutions

Rhodamine 123, Hoechst 33342, doxorubicin and verapamil were obtained from Sigma-Aldrich (St. Louis, MO, USA). All reagents’ solutions were freshly prepared before experiment by dissolving in sterile deionized water. Celastrol with purity more than 98% was purchased from Cayman Chemical Company (Ann Arbor, MI, USA). Celastrol was dissolved with dimethylsulfoxide, DMSO, (Sigma-Aldrich, St. Louis, MO, USA) as 10mM stock solution, stored at −20°C.

### Cytometry and cytometric data analysis software

In all tests performed, the samples of cells were analyzed with CyFlow® SPACE flow cytometer (Sysmex-Partec, Görlitz, Germany) with laser excitation and BP filters recommended for a specific test method, as will be detailed below in the description of procedures for each test performed. The results were analyzed with FCS express 4 flow software (De Novo Software, Glendale, CA, USA).

### Cytotoxicity and apoptosis assessments using Annexin V-FITC and IP staining

The FITC Annexin V Apoptosis Detection Kit I BD Biosciences (Franklin Lakes, NJ, USA) was used for discriminating between necrotic, early and late apoptotic cells by the means of Annexin V-FITC and PI labelling. LOVO/DX cells (5 × 10^5^) cells were incubated (4 hours) in 24-well plate in the absence (control) and presence of various celastrol concentrations (37°C, 5% CO_2_). Following incubation, cells were removed using TrypLE™ Express solution and washed with cold HBSS (Hank's Balanced Salt Solution; Lonza, Basel, Switzerland). The cell samples were resuspended in 100μl of ice-cold 1X binding buffer and stained with 5μl of Annexin V-FITC and 5μl of PI for 10 min in the dark at room temperature. Samples were immediately analyzed with CyFlow® SPACE flow cytometer applying laser excitation 488nm (50mW) and filters: 536/40(BP) for Annexin V-FITC and 675/20 (BP) for PI.

### Intracellular doxorubicin and rhodamine 123 accumulation studies

Accumulation of rhodamine 123 [55] and doxorubicin [56] in cells was evaluated by flow cytometry. LOVO/DX cells (1×10^6^) were resuspended in 2ml of pre-warmed HBSS in plastic Falcon tubes and celastrol solution was added to the samples (to the final concentrations of 0.15-20μM). After short (5min) incubation with celastrol, rhodamine 123 (5μM final) or doxorubicin (3μM final) solution were added to all samples, which were further incubated for 1hour at 37°C. Afterwards, cells were washed with ice cold HBSS (4°C), cells pellet was resuspended in 1ml of ice cold HBSS and immediately analyzed with CyFlow® SPACE flow cytometer with the laser excitation was 488nm (50mW) and the filters used were 536/40 (BP) for rhodamine 123 and 590/50 (BP) for doxorubicin fluorescence measurements.

### The UIC2 reactivity shift assay

The UIC2 shift assay was performed using mouse anti-human CD243:Alexa Fluor ®488 monoclonal antibodies (AbD Serotec/BIO RAD, Oxford, UK). The protocol used in the study have been described in detail by Park S.W. et al. [43]. Briefly, cells (1×10^6^) were resuspended in 2ml of pre-warmed PBS supplemented with 2% FBS and placed into plastic Falcon tubes. The cells were incubated 10 minutes at 37°C in a humidified atmosphere with 5% CO_2_. Then, 20μl aliquots of celastrol solution were added and cells were incubated for another 10 minutes. After one washing step, cells were resuspended in 100μl of pre-warmed HBSS supplemented with 2% FBS and mixed with 2μl of celastrol solution and 10μl of antibody. The cells were further incubated for 15 minutes at 37°C in a humidified atmosphere with 5% CO_2_. After incubation, cells were washed and resuspended in 1ml of ice cold HBSS and the samples were immediately analyzed with CyFlow® SPACE cytometer with laser excitation 488nm (50mW) and filter 536/40(BP) for Alexa Fluor®488.

### SP cell size estimation with Hoechst 33342 exclusion assay

The SP analysis was performed according to the classical SP protocol described by Goodel et all. [57, 58]. LOVO/DX cells were suspended in a mixture of pre-warm HBSS/ 2% FBS to the final cell density 1×10^6^ cells/ml. Cells suspension was placed into plastic Falcon tubes and celastrol solution was added to the samples (to the final concentrations 0.15-20μM). Verapamil at final concentration of 50μM was used as positive control. After 5-10 minutes of incubation, Hoechst 33342 water solution (1mg/ml) was added to yield a concentration of 5μg/ml. The samples were then incubated at 37°C for 90 minutes with shaking every 30 minutes. The cells were washed and resuspended with ice-cold HBSS. To exclude death cells from analysis, the cells were stained with Annexin V-FITC and propidium iodide (FITC Annexin V Apoptosis Detection Kit I BD Biosciences). The samples were kept on ice during flow cytometric analysis using CyFlow® SPACE cytometer. The Hoechst dye was excited with UV laser (λ=365nm) and its fluorescence was measured with a 455/50 BP filter (Hoechst blue) and a 630 LP filter (Hoechst red). In parallel, (50mW; λ=488nm) laser and the fluorescence filters: 536/40(BP) for Annexin V-FITC and 675/20 (BP) for PI were used.

### Identification of aldehyde dehydrogenase 1 (ALDH 1) expressing cells

ALDH1 labeling was performed using the ALDEFLUOR™ Kit according to the manufacturer's instructions (Stem Cell Technology, Vancouver, Canada). For combined tagging of SP cells subpopulation and ALDH, LOVO/DX cells were first stained with Hoechst 33342, as described previously, and then labeled with activated ALDEFLUOR™ reagent, BODIPY-aminoacetaldehyd. Briefly, LOVO/DX cells (1×106/ml) were pre-incubated with celastrol (1 and 10μM) for 30 minutes in plastic Falcon tubes, followed by 90 minutes incubation with Hoechst 33342 (5μg/ml, final concentration). The cells were washed, resuspended in ALDEFLUOR™ assay buffer and stained with activated ALDEFLUOR™ reagent. The control samples containing diethylaminobenzaldehyde (DEAB), a specific inhibitor of ALDH, were used to control background fluorescence. After ALDEFLUOR™ staining, the cells were centrifuged, resuspended in ALDEFLUOR™ assay buffer, and analyzed on CyFlow® SPACE flow cytometer applying two lasers for fluorescence excitation: λ=488nm (50mW) and λ=365nm (16mW) and the filters for fluorescence measurement: 536/40 (BP) for ALDH fluorescence measurement and 455/50 (BP) and 630 (LP) for Hoechst 33342 (SP analysis).

### Statistical analysis

Statistical analysis of the results: paired *t* test, Kruskal-Wallis test with Dunn's post hoc test and Spearman's rank correlation test as well as regression analysis were made using the GraphPad Prism software, version 7.02 (GraphPad Software, La Jolla, CA, USA).

## Abbreviations

P-gp: glycoprotein P
ABCB1: ATP binding cassette subfamily B member 1
MDR 1: multidrug resistance protein 1
CSCs: cancer stem cells
BCRP: breast cancer resistance protein
PI: propidium iodide
DOX: doxorubicin
Rod-123: rhodamine-123
VER: Verapamil
Ho: Hoechst 33342
SP: Side Population

## References

[1] Ricci-Vitiani L, Pagliuca A, Palio E, Zeuner A, De Maria R (2008). Colon cancer stem cells. Gut.

[2] Hammond WA, Swaika A, Mody K (2016). Pharmacologic resistance in colorectal cancer: a review. Ther Adv Med Oncol.

[3] Longley DB, Johnston PG (2005). Molecular mechanisms of drug resistance. J Pathol.

[4] Taniguchi H, Moriya C, Igarashi H, Saitoh A, Yamamoto H, Adachi Y, Imai K (2016). Cancer stem cells in human gastrointestinal cancer. Cancer Sci.

[5] Alisi A, Cho WC, Locatelli F, Fruci D (2013). Multidrug resistance and cancer stem cells in neuroblastoma and hepatoblastoma. Int J Mol Sci.

[6] Moitra K, Lou H, Dean M (2011). Multidrug efflux pumps and cancer stem cells: Insights into multidrug resistance and therapeutic development. Clin Pharmacol Ther.

[7] Vermeulen L, de Sousa e Melo F, Richel DJ, Medema JP (2012). The developing cancer stem-cell model: Clinical challenges and opportunities. Lancet Oncol.

[8] Clevers H (2011). The cancer stem cell: premises, promises and challenges. Nat Med.

[9] Garza-Treviño EN, Said-Fernández SL, Martínez-Rodríguez HG (2015). Understanding the colon cancer stem cells and perspectives on treatment. Cancer Cell Int.

[10] Kramer R, Weber TK, Morse B, Arceci R, Staniunas R, Steele G, Summerhayes IC (1993). Constitutive expression of multidrug resistance in human colorectal tumours and cell lines. Br J Cancer.

[11] Gottesman MM, Fojo T, Bates SE (2002). Multidrug resistance in cancer: role of ATP-dependent transporters. Nat Rev Cancer.

[12] Wesołowska O, Wiśniewski J, Środa-Pomianek K, Bielawska-Pohl A, Paprocka M, Duś D, Duarte N, Ferreira MJ, Michalak K (2012). Multidrug resistance reversal and apoptosis induction in human colon cancer cells by some flavonoids present in citrus plants. J Nat Prod.

[13] Yan XJ, Gong LH, Zheng FY, Cheng KJ, Chen ZS, Shi Z (2014). Triterpenoids as reversal agents for anticancer drug resistance treatment. Drug Discov Today.

[14] Di C, Zhao Y (2015). Multiple drug resistance due to resistance to stem cells and stem cell treatment progress in cancer (Review). Exp Ther Med.

[15] Riccioni R, Dupuis ML, Bernabei M, Petrucci E, Pasquini L, Mariani G, Cianfriglia M, Testa U (2010). The cancer stem cell selective inhibitor salinomycin is a p-glycoprotein inhibitor. Blood Cells Mol Dis.

[16] Kanadaswami C, Lee LT, Lee PP, Hwang JJ, Ke FC, Huang YT, Lee MT (2005). The antitumor activities of flavonoids. *In Vivo*.

[17] Karikas GA (2010). Anticancer and chemopreventing natural products: Some biochemical and therapeutic aspects. J BUON.

[18] Allison AC, Cacabelos R, Lombardi VR, Álvarez XA, Vigo C (2001). Celastrol, a potent antioxidant and anti-inflammatory drug, as a possible treatment for Alzheimer’s disease. Prog Neuropsychopharmacol Biol Psychiatry.

[19] Kim DH, Shin EK, Kim YH, Lee BW, Jun JG, Park JH, Kim JK (2009). Suppression of inflammatory responses by celastrol, a quinone methide triterpenoid isolated from Celastrus regelii. Eur J Clin Invest.

[20] Venkatesha SH, Yu H, Rajaiah R, Tong L, Moudgil KD (2011). Celastrus-derived celastrol suppresses autoimmune arthritis by modulating antigen-induced cellular and humoral effector responses. J Biol Chem.

[21] Pinna GF, Fiorucci M, Reimund JM, Taquet N, Arondel Y, Muller CD (2004). Celastrol inhibits pro-inflammatory cytokine secretion in Crohn’s disease biopsies. Biochem Biophys Res Commun.

[22] Jung HW, Chung YS, Kim YS, Park YK (2007). Celastrol inhibits production of nitric oxide and proinflammatory cytokines through MAPK signal transduction and NF-κB in LPS-stimulated BV-2 microglial cells. Exp Mol Med.

[23] Liu Z, Ma L, Zhou GB (2011). The main anticancer bullets of the chinese medicinal herb, thunder god vine. Molecules.

[24] Venkatesha SH, Moudgil KD (2016). Celastrol and its role in controlling chronic diseases. Adv Exp Med Biol.

[25] Tan KW, Li Y, Paxton JW, Birch NP, Scheepens A (2013). Identification of novel dietary phytochemicals inhibiting the efflux transporter breast cancer resistance protein (BCRP/ABCG2). Food Chem.

[26] Davenport A, Frezza M, Shen M, Yubin G, Huo C, Chan TH, Dou QP (2010). Celastrol and an EGCG pro-drug exhibit potent chemosensitizing activity in human leukemia cells. Int J Mol Med.

[27] Barker E, Letterio JJ, Tochtrop GP (2013). Celastrol shows chemopreventive properties in an inflammatory driven model for colon cancer via induction of Nrf2 transcription. Cancer Res.

[28] Chen M, Rose AE, Doudican N, Osman I, Orlow SJ (2009). Celastrol synergistically enhances temozolomide cytotoxicity in melanoma cells. Mol Cancer Res.

[29] Kannaiyan R, Hay HS, Rajendran P, Li F, Shanmugam MK, Vali S, Abbasi T, Kapoor S, Sharma A, Kumar AP, Chng WJ, Sethi G (2011). Celastrol inhibits proliferation and induces chemosensitization through down-regulation of NF-κB and STAT3 regulated gene products in multiple myeloma cells. Br J Pharmacol.

[30] Zuo H, Yin Z, Baogen M, Yaping D, Yulong L, Zuo C (2011). The Experimental studies of celastrol on the reversal of multidrug resistance in K562/A02 cell line. Pract J Cancer.

[31] Mou H, Zheng Y, Zhao P, Bao H, Fang W, Xu N (2011). Celastrol induces apoptosis in non-small-cell lung cancer A549 cells through activation of mitochondria- and Fas/FasL-mediated pathways. Toxicol *In Vitro*.

[32] Kroemer G, Galluzzi L, Vandenabeele P, Abrams J, Alnemri E, Baehrecke E, Blagosklonny M, El-Deiry W, Golstein P, Green D, Hengartner M, Knight R, Kumar S (2009). Classification of cell death 2009. Cell Death Differ.

[33] Kannaiyan R, Manu KA, Chen L, Li F, Rajendran P, Subramaniam A, Lam P, Kumar AP, Sethi G (2011). Celastrol inhibits tumor cell proliferation and promotes apoptosis through the activation of c-Jun N-terminal kinase and suppression of PI3 K/Akt signaling pathways. Apoptosis.

[34] Yoon MJ, Lee AR, Jeong SA, Kim YS, Kim JY, Kwon YJ, Choi KS (2014). Release of Ca2+ from the endoplasmic reticulum and its subsequent influx into mitochondria trigger celastrol-induced paraptosis in cancer cells. Oncotarget.

[35] Wang WB, Feng LX, Yue QX, Wu WY, Guan SH, Jiang BH, Yang M, Liu X, Guo DA (2012). Paraptosis accompanied by autophagy and apoptosis was induced by celastrol, a natural compound with influence on proteasome, ER stress and Hsp90. J Cell Physiol.

[36] Li HY, Zhang J, Sun LL, Li BH, Gao HL, Xie T, Zhang N, Ye ZM (2015). Celastrol induces apoptosis and autophagy via the ROS/JNK signaling pathway in human osteosarcoma cells: an *in vitro* and *in vivo* study. Cell Death Dis.

[37] Kulbacka J, Saczko J, Chwiłkowska A (2008). [Colon cancer- characteristic and treatment resistance]. [Article in Polish]. Onkol Prak Klin.

[38] Dietrich CG, Geier A, Oude Elferink RP (2003). ABC of oral bioavailability: transporters as gatekeepers in the gut. Gut.

[39] Abdallah HM, Al-Abd AM, El-Dine RS, El-Halawany AM (2015). P-glycoprotein inhibitors of natural origin as potential tumor chemo-sensitizers : A review. J Adv Res.

[40] Yoshida N, Koizumi M, Adachi I, Kawakami J (2006). Inhibition of P-glycoprotein-mediated transport by terpenoids contained in herbal medicines and natural products. Food Chem Toxicol.

[41] Muñoz-Martínez F, Lu P, Cortés-Selva F, Pérez-Victoria JM, Jiménez IA, Ravelo ÁG, Sharom FJ, Gamarro F, Castanys S (2004). Celastraceae sesquiterpenes as a new class of modulators that bind specifically to human P-glycoprotein and reverse cellular multidrug resistance. Cancer Res.

[42] Muñoz-Martínez F, Reyes CP, Pérez-Lomas AL, Jiménez IA, Gamarro F, Castanys S (2006). Insights into the molecular mechanism of action of Celastraceae sesquiterpenes as specific, non-transported inhibitors of human P-glycoprotein. Biochim Biophys Acta.

[43] Park SW, Lomri N, Simeoni LA, Fruehauf JP, Mechetner E (2003). Analysis of P-glycoprotein-mediated membrane transport in human peripheral blood lymphocytes using the UIC2 shift assay. Cytometry A.

[44] Mechetner EB, Schott B, Morse BS, Stein WD, Druley T, Davis KA, Tsuruo T, Roninson IB (1997). P-glycoprotein function involves conformational transitions detectable by differential immunoreactivity. Proc Natl Acad Sci U S A.

[45] Maki N, Hafkemeyer P, Dey S (2003). Allosteric modulation of human P-glycoprotein. Inhibition of transport by preventing substrate translocation and dissociation. J Biol Chem.

[46] Visvader JE, Lindeman GJ (2008). Cancer stem cells in solid tumours: accumulating evidence and unresolved questions. Nat Rev Cancer.

[47] Chen K, Huang Y, Chen J (2013). Understanding and targeting cancer stem cells: therapeutic implications and challenges. Acta Pharmacol Sin.

[48] Abdullah LN, Chow EK (2013). Mechanisms of chemoresistance in cancer stem cells. Clin Transl Med.

[49] Christgen M, Ballmaier M, Bruchhardt H, Wasielewski R, Kreipe H, Lehmann U (2007). Identification of a distinct side population of cancer cells in the Cal-51 human breast carcinoma cell line. Mol Cell Biochem.

[50] Chiba T, Kita K, Zheng YW, Yokosuka O, Saisho H, Iwama A, Nakauchi H, Taniguchi H (2006). Side population purified from hepatocellular carcinoma cells harbors cancer stem cell-like properties. Hepatology.

[51] Haraguchi N, Inoue H, Tanaka F, Mimori K, Utsunomiya T, Sasaki A, Mori M (2006). Cancer stem cells in human gastrointestinal cancers. Hum Cell.

[52] Kondo T (2007). Stem cell-like cancer cells in cancer cell lines. Cancer Biomark.

[53] Boesch M, Zeimet AG, Fiegl H, Wolf B, Huber J, Klocker H, Gastl G, Sopper S, Wolf D (2016). High prevalence of side population in human cancer cell lines. Oncoscience.

[54] Donnenberg AD, Hicks JB, Wigler M, Donnenberg VS (2013). The Cancer stem cell: Cell type or cell state?. Cytometry A.

[55] Pétriz J, García-López J (1997). Flow cytometric analysis of P-glycoprotein function using rhodamine 123. Leukemia.

[56] Luk CK, Tannock IF (1989). Flow cytometric analysis of doxorubicin accumulation in cells from human and rodent cell lines. J Natl Cancer Inst.

[57] Goodell MA, Brose K, Paradis G, Conner AS, Mulligan RC (1996). Isolation and functional properties of murine hematopoietic stem cells that are replicating *in vivo*. J Exp Med.

[58] Lin KK, Goodell MA (2006). Purification of hematopoietic stem cells using the side population. Methods Enzymol.

[59] Lee JH, Koo TH, Yoon H, Jung HS, Jin HZ, Lee K, Hong YS, Lee JJ (2006). Inhibition of NF-κB activation through targeting IκB kinase by celastrol, a quinone methide triterpenoid. Biochem Pharmacol.

[60] Jin HZ, Hwang BY, Kim HS, Lee JH, Kim YH, Lee JJ (2002). Antiinflammatory constituents of Celastrus orbiculatus inhibits the NF-κB activation and NO production. J Nat Prod.

[61] Sethi G, Ahn KS, Pandey MK, Aggarwal BB (2007). Celastrol, a novel triterpene potentiates TNF induced apoptosis and suppresses invasion of tumor cells by inhibiting NF-κB-regulated gene product and NF-κBactivation. Blood.

[62] Bentires-Alj M, Barbu V, Fillet M, Chariot A, Relic B, Jacobs G, Merville MP, Bours V (2003). NκB transcription factor induces drug resistance through MDR1 expression in cancer cells. Oncogene.

[63] Kuo MT, Liu Z, Lin-Lee YC, Tatebe S, Mills GB, Unate H (2002). Induction of human MDR1 gene expression by 2-acetylaminofluorene is mediated by effectors of the phosphoinositide 3-kinase pathway that activate NF-kappaB signaling. Oncogene.

[64] Kim HG, Hien TT, Han EH, Hwang YP, Choi JH, Kang KW, Kwon KI, Kim BH, Kim SK, Song GY, Jeong TC, Jeong HG (2011). Metformin inhibits P-glycoprotein expression via the NF-κB pathway and CRE transcriptional activity through AMPK acvtivation. Br J Pharmacol.

[65] Godwin P, Baird AM, Heavey S, Barr MP, O'Byme KJ, Gately K (2013). Targeting nuclear factor kappa B to overcome resistance to chemotherapy. Front Oncol.

[66] Li F, Sethi G (2010). Targeting transcription factor NF-κB to overcome chemoresistance and radioresistance in cancer therapy. Biochim Biophys Acta.

[67] Hoesel B, Schmid JA (2013). The complexity of NF-κB signaling in inflammation and cancer. Mol Cancer.

[68] Nakanishi C, Toi M (2005). Nuclear factor-kappa B inhibitrors as sensitizers to anticancer drugs. Nat Rev Cancer.

[69] Cusack JC, Liu R, Baldwin AS (1999). NF-κB and chemoresistance: potentiation of cancer chermotherapy via inhibition of NF-κB. Drug Resist Updat.

[70] House CD, Jordan E, Hernandez L (2017). NF-κB promotes ovarian tumotrigenesis via classical pathways supporting proliferative cancer cells and alternative pathways supporting ALDH+ cancer stem-like cells. Cancer Res.

[71] Vazquez-Santillan K, Melendez-Zajgla J, Jimenez-Hernandez LE, Gaytan-Cervantes J, Muñoz-Galindo L, Piña-Sanchez P, Martinez-Ruiz G, Torres J, Garcia-Lopez P, Gonzalez-Torres C, Ruiz V, Avila-Moreno F, Velasco-Velazquez M NF-kappaB-inducing kinase regulates stem cell phenotype in greast cancer. Sci Rep.

